# Telomerase activity assay for the diagnosis of malignant pleural effusion: A meta-analysis

**DOI:** 10.3892/etm.2012.623

**Published:** 2012-06-26

**Authors:** YONG-CHUN SHEN, ZHEN-NI CHEN, TING YANG, LEI CHEN, TAO WANG, FU-QIANG WEN, QUN YI

**Affiliations:** 1 Division of Pulmonary Diseases, State Key Laboratory of Biotherapy of China, Department of Respiratory Medicine, West China Hospital of Sichuan University;; 2 Department of Health Education Institution, West China School of Public Health, Chengdu, Sichuan 610041, P.R. China

**Keywords:** malignant pleural effusion, telomerase, meta-analysis

## Abstract

The telomerase activity assay has been established for the detection of malignant pleural effusion (MPE), however, the overall diagnostic accuracy of the telomerase activity assay for MPE remains unclear. We performed a systematic search in the Pubmed, Embase and Cochrane databases to identify published studies that have evaluated the diagnostic role of the telomerase activity assay for MPE. Sensitivity, specificity and other measures of accuracy of the telomerase activity assay in the diagnosis of MPE were pooled using the random effects models. A summary receiver operating characteristic (SROC) curve was used to summarize overall test performance. A total of eight studies met the inclusion criteria for the meta-analysis. The pooled sensitivity and specificity for diagnosing MPE were 0.76 [95% confidence intervals (CI), 0.72–0.80] and 0.87 (95% CI, 0.83–0.91), respectively. The positive likelihood ratio was 5.19 (95% CI, 2.36–11.42), the negative likelihood ratio was 0.25 (95% CI, 0.11–0.53) and the diagnostic odds ratio was 23.18 (95% CI, 6.11–87.83). The area under the SROC curve was 0.92. The telomerase activity assay plays a role in the diagnosis of MPE with a relatively high specificity. The results of a telomerase activity assay should be interpreted together with the combination of other test results and clinical findings.

## Introduction

Pleural effusion is a frequent complication in patients with cardiac failure, pneumonia, tuberculosis and neoplasms (1). Malignancy is one of the most significant causes of pleural effusion and more than 90% of malignant pleural effusions (MPEs) are caused by metastatic diseases (2). It is necessary to elucidate their etiologies, yet to differentiate MPE from benign pleural effusion remains a clinical challenge (3). Initial diagnostic methods include cytological, histological, biochemical and thoracocentesis examinations. However, the overall sensitivity of cytological examination is only 60% (3), thoracoscopic pleural biopsy or image-guided percutaneous pleural biopsy provides a relative high sensitivity, but may not be available in all hospitals or well-tolerated (4).

A series of tumor markers have been well-studied for their ability to improve the diagnosis of MPE. Three published studies have investigated the diagnostic value of the pleural vascular endothelial growth factor, carcinoembryonic antigen (CEA), carbohydrate antigens (CA) 125, 15–3 and 19–9, and CYFRA 21–1 in MPE but have failed to identify a reliable tumor marker with both high sensitivity and high specificity. The authors did not recommend using one tumor marker alone for the diagnosis of MPE (5–7). Therefore it is imperative to find a new diagnostic tool to facilitate diagnostic accuracy.

Telomerase is a specialized reverse transcriptase that adds TTAGGG repeats to the telomeric ends of chromosomal DNA to maintain the telomeric length (8). The expression of telomerase activity has been shown to be correlated with telomeric length (9). Telomerase is active in many types of human cancers but is not detectable in most normal somatic cells (10,11). Therefore telomerase activity may be a universal and specific marker for diagnosing a wide variety of cancers (12). Several studies have shown that the measurement of telomerase activity may be a useful and noninvasive method to detect malignancy in body fluid, particularly when used in combination with conventional cytological examination (13,14). A number of studies on the potential diagnostic role of telomerase activity assay in MPE have been published and have reported varying results. The present meta-analysis aimed to establish the overall accuracy of the telomerase activity assay in the diagnosis of MPE.

## Materials and methods

### Meta-analysis

The present meta-analysis was performed according to the guidelines of the Preferred Reporting Items for Systematic Reviews and Meta-Analysis (PRISMA) statement and with methods recommended by the Cochrane Diagnostic Test Accuracy Working Group (15,16).

### Search strategy and study selection

To identify studies that have evaluated the evidence of using telomerase activity in order to diagnose MPE, we performed a search of the Pubmed (Medline), Embase and Cochrane databases up to March 15, 2012, using the key words ‘pleural effusion’, ‘malignant pleural effusions’ and ‘telomerase activity’. Although no language restrictions were imposed on the search criteria, only English-and Chinese-language publications concerning human studies were included in the present meta-analysis. In addition, a manual search of the reference lists of eligible papers was also conducted.

Conference abstracts were excluded due to the limited data provided. A study was included in the present meta-analysis if it provided data on both sensitivity and specificity of pleural telomerase activity for the diagnosis of MPE. Studies with fewer than 10 patients were excluded to avoid selection bias. Two authors (Y-C Shen and Z-N Chen) independently screened the articles for inclusion. Disagreements between authors were resolved by consensus.

### Data extraction and quality assessment

The final set of articles was assessed independently by two reviewers (Y-C Shen and Z-N Chen). The data retrieved from the reports included author, publication year, patient source, test method, sensitivity and specificity data, and methodological quality. When the same patients were reported in several studies, only the most informative article was included to avoid duplication of information. The methodological quality of studies was evaluated by a QUADAS tool (Quality Assessment for Studies of Diagnostic Accuracy, an evidence based quality assessment tool to be used in systematic reviews of diagnostic accuracy studies, maximum score 14) (17).

### Statistical analyses

The standard methods recommended for the meta-analyses of diagnostic accuracy studies were used in the present study (18). The following measures of test accuracy were calculated for each study: sensitivity, specificity, positive likelihood ratio (PLR), negative likelihood ratio (NLR) and diagnostic odds ratio (DOR), together with their 95% confidence interval (CI). The present meta-analysis was based on a summary receiver operating characteristic (SROC) curve, and the sensitivity and specificity for the single test threshold identified for each study was used to plot an SROC curve (19). A random-effects meta-analysis was performed in order to account for the differences between study variability for each study. The Spearman's rank correlation was performed as a test for threshold effect. Chi-squared and Fisher's exact tests were used to detect statistically significant heterogeneity across studies. Since publication bias is of concern for meta-analyses of diagnostic studies, we tested for the potential presence of this bias using Deeks funnel plots (20). All analyses were performed using two statistical software programs (Meta-DiSc for Windows; XI Cochrane Colloquium, Barcelona, Spain and Stata, version 11; Stata Corporation, College Station, TX, USA). All statistical tests were two-sided and P<0.05 was considered to indicate a statistically significant result.

## Results

### Study exclusion criteria

After independent review, eight studies with 678 samples on the use of telomerase activity in patients with MPE were considered eligible for inclusion in the present meta-analysis (21–28). The major reasons for excluding the other studies were as follows: non-diagnostic studies or studies cannot reconstruct the diagnostic 2 by 2 table; limited samples, or mixed with other serous effusions and duplicated studies. The clinical summary of these studies, along with the QUADAS scores, are outlined in Table I.

### Study characteristics and quality report

There were five English and three Chinese articles. The average sample size in the eight studies was 85 (range from 28 to 144) and the samples included 375 patients with MPE and 303 non-MPE. Telomerase activity was measured by a PCR-based or PCR-ELISA method. The current gold standards, cytological and histological examinations, are highly reliable methods to identify MPE. The quality of the eight studies was generally high with six studies having QUADAS scores ≥10.

### Diagnostic accuracy for MPE

**Figure 1:**
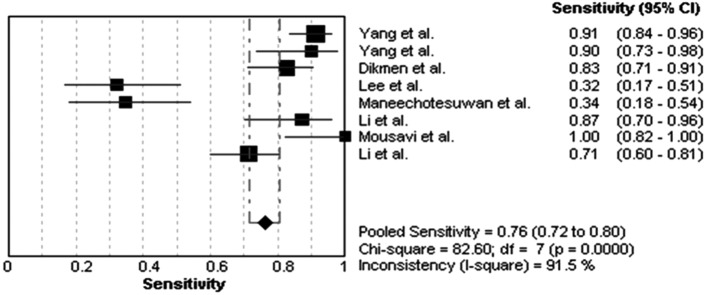
Forest plots of sensitivity for the telomerase activity assay. The point estimates of sensitivity from each study are shown as solid squares. Error bars indicate 95% confidence intervals. CI, Confidence intervals.

**Figure 2:**
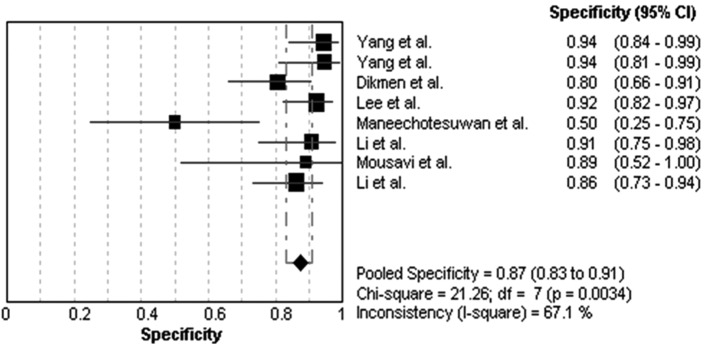
Forest plots of specificity for the telomerase activity assay. The point estimates of specificity from each study are shown as solid squares. Error bars indicate 95% confidence intervals. CI, Confidence intervals.

The heterogeneity analysis revealed I^2^-values of 91.5% for sensitivity and 67.1% for specificity. The existence of significant heterogeneity occurred in the eight studies, thus the random effects model approach was selected for the present meta-analysis. The forest plots of the sensitivity and specificity for eight telomerase activity assays in diagnosing MPE are shown in Figs. 1 and 2, respectively. The pooled sensitivity was 0.76 (95% CI, 0.72–0.80), specificity was 0.87 (95% CI, 0.83–0.91). The PLR was 5.19 (95% CI, 2.36–11.42), the NLR was 0.25 (95% CI, 0.11–0.53) and the DOR was 23.18 (95% CI, 6.11–87.83).

**Figure 3:**
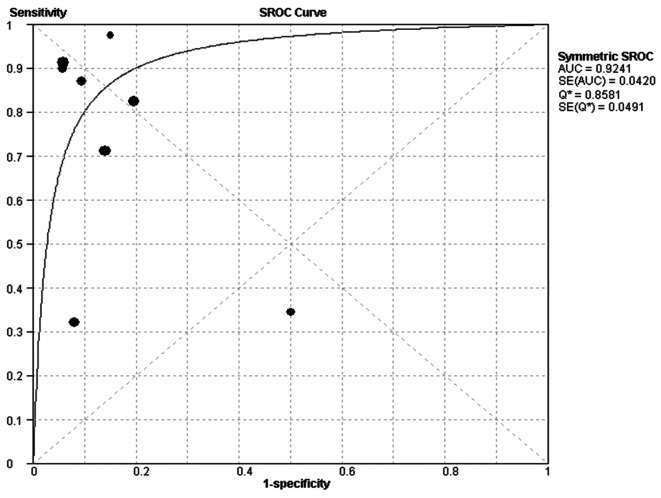
Summary receiver operating characteristic (SROC) curve for telomerase activity assay. The size of each solid circle represents the size of each study included in the present meta-analysis. The regression SROC curve indicates the overall diagnostic accuracy. AUC, area under curve; SE, standard error.

The SROC curve plotting the true-positive against the false-positive rates of individual studies is shown in Fig. 3. The area under curve (AUC) was 0.92, indicating that the level of overall accuracy was high.

### Publication bias

**Figure 4:**
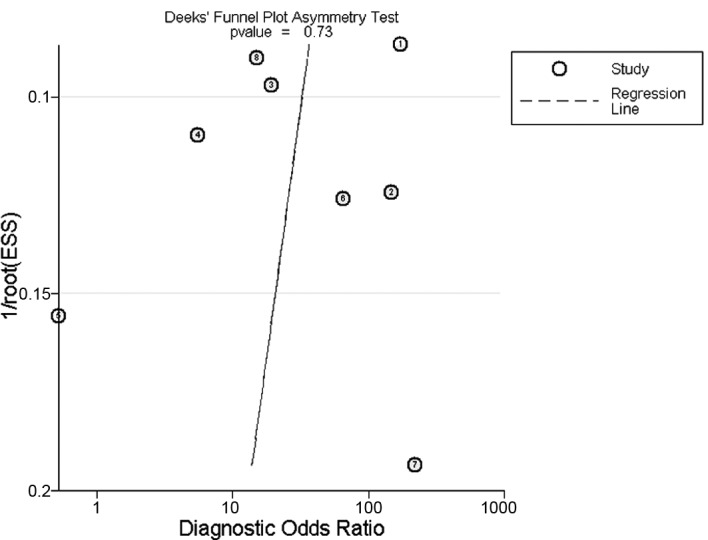
Linear regression test of funnel plot asymmetry. The statistically insignificant value (P= 0.73) for the slope coefficient suggests symmetry in the data and a low likelihood of publication bias. The number in the circle means the study number as listed in Table I.

The Deeks funnel plot asymmetry test was used to evaluate potential publication bias; the statistically insignificant value (P=0.73) for the slope coefficient suggests symmetry in the data and a low likelihood of publication bias (Fig. 4)

In addition, we did not perform a meta-regression analysis with QUADAS scores to assess the effect of study quality on relative DOR of telomerase activity in the diagnosis of MPE due to the limited numbers of studies included. For the same reason, we could not explore whether or not the study design including blinded, cross-sectional, consecutive/random and prospective design affected diagnostic accuracy.

## Discussion

The present meta-analysis evaluated the overall diagnostic role of pleural telomerase activity in MPE. To the best of our knowledge, this is the first meta-analysis to assess the diagnostic role of telomerase activity in MPE. We found a summary AUC of 0.92, a summary estimate of 0.76 for sensitivity and 0.87 for specificity. It appears that telomerase activity examination plays a valuable role in the diagnosis of MPE. It may be more favor in confirming MPE.

The SROC curve has been recommended to represent the performance of a diagnostic test, and shows the trade-off between sensitivity and specificity based on data from a meta-analysis (29,30). The AUC and an index Q^*^ are recognized as potentially useful summaries of the curve. We used the Q-value, intersection point of the SROC curve with a diagonal line from the left upper corner to the right lower corner of the ROC space, which corresponds to the highest common value of sensitivity and specificity for the test and represents an overall measure of the discriminatory power of a test. In the present study, the Q-value was 0.86, demonstrating that the maximum joint sensitivity and specificity was 0.86. The AUC also measures the overall accuracy of diagnostic studies. If the AUC is 1, then the telomerase activity test differentiates perfectly between MPE and non-MPE subjects. An AUC of greater than 0.9 indicates high diagnostic accuracy. In our meta-analysis, the AUC was 0.92, suggesting the level of overall accuracy was high.

The DOR is a single indicator of test accuracy that combines the data from sensitivity and specificity into a single number (31). It is the ratio of the odds of positive test results in the diseased, relative to the odds of positive test results in the non-diseased. The value of a DOR ranges from 0 to infinity, with higher values indicating an improved discriminatory test performance. In the present meta-analysis, the mean DOR was 23.18, suggesting that the telomerase activity assays appeared to be useful in the diagnosis of MPE. Since the SROC curve and DOR are not easy to interpret and use in clinical practice, likelihood ratios are considered more clinically meaningful (32). The PLR was 5.19, indicating that patients with MPE have an approximate 5-fold higher chance of being telomerase activity assay-positive compared with patients without MPE. However, the PLR was not high enough for making a definitive clinical decision. The NLR was found to be 0.25, which means if the telomerase activity assay result was negative, the probability that this patient has MPE is 25%, which is not low enough to exclude MPE.

Our meta-analysis suggests that telomerase activity determination plays a valuable role in diagnosing MPE, especially in the confirmation of MPE. The reported sensitivities varied among studies, ranging from 0.32 to 1.00. As Lee (24) suggested that the ELISA-based telomerase activity assay is relatively insensitive, it is unsuitable as a routine screening tool for MPE. False-positive telomerase activity due to lymphocytic contamination may weaken the diagnostic value for MPE in a tuberculosis-endemic area (24). The combination with other markers for pleural effusion would aid in increasing the sensitivity. For instance, the combination of telomerase activity with CEA or CYFRA21-1 was found to provide a high sensitivity of 0.93 and 0.90, respectively (26,28). The further development of the method of telomerase activity measurement may increase diagnostic accuracy for MPE. Hansson *et al* (33) reported that telomerase activity measurement *in situ* based on cytospins from fresh cytological material correlated well to final diagnoses and could differentiate between MPE and non-MPE cases. It was suggested that this method may improve the diagnostic accuracy for MPE (33,34). Cytological and/or histological examinations remain the traditional method for diagnosing MPE, however, pathologists do not recommend a diagnosis solely based on cytological samples due to the high risk of diagnostic error. In addition, the invasive thoracoscopy may not be available in all hospitals (3,4). Thus, the importance of the telomerase activity test is not only to provide high specificity with which to confirm the diagnosis of MPE, but also to guide the inclusion of patients who may benefit from further invasive procedures.

The present meta-analysis had several limitations. Although we strengthened the present meta-analysis comprehensive search strategy and data extraction, only eight studies with 678 samples were included. The evidence generated from the limited studies and samples may limit the interpretation of the meta-analysis results. Secondly, we excluded conference abstracts and non-English or non-Chinese publications, which may have led to publication bias (35), which may also be introduced by inflation of diagnostic accuracy estimates since studies that report positive findings are more likely to be accepted for publication. However, there was no publication bias reported in the present meta-analysis due to the limited number of studies included. We did not use QUADAS scores to perform the meta-regression analysis or explore whether or not the study design affected diagnostic accuracy.

Based on the evidence compiled in this meta-analysis, telomerase activity measurement plays a role in the diagnosis of MPE, and it is likely to be a useful diagnostic tool for confirming MPE. The results of telomerase activity assays should be interpreted in parallel with clinical findings and the results of conventional tests. Studies with larger sample sizes and improved telomerase activity examination methods are still required to determine the diagnostic performance of the telomerase activity assay for MPE.

## Figures and Tables

**Table I:** Clinical summary of the included studies.

	Sample size								
Study, year (ref.)	MPE	Non-MPE	Standard	Method	TP	FP	FN	TN	QUADAS
1. Yang *et al*, 1998 (21)	92	52	Histology/Cytology	PCR	84	3	8	49	12
2. Yang *et al*, 2001 (22)	30	35	Histology	PCR-ELISA	27	2	3	33	11
3. Dikmen *et al*, 2003 (23)	63	46	Histology/Cytology	PCR	52	9	11	37	10
4. Lee *et al*, 2005 (24)	31	63	Histology/Cytology	PCR-ELISA	10	5	21	58	11
5. Maneechotesuwan *et al*, 2006 (25)	29	16	Histology/Cytology	PCR	10	8	19	8	10
6. Li *et al*, 2008 (26)	31	32	Histology/Cytology	PCR-ELISA	27	3	4	29	9
7. Mousavi *et al*, 2009 (27)	19	9	Histology/Cytology	PCR-ELISA	19	1	0	8	9
8. Li *et al*, 2010 (28)	80	50	Histology/Cytology	PCR-ELISA	57	7	23	43	10

MPE, malignant pleural effusion. TP, true positive; FP, false positive; FN, false negative; TN, true negative; QUADAS, Quality Assessment for Studies of Diagnostic Accuracy; PCR, polymerase chain reaction; ELISA, enzyme linked immunosorbent assay.
